# *HcZnT2* is a highly mycorrhiza-induced zinc transporter from *Hebeloma cylindrosporum* in association with pine

**DOI:** 10.3389/fpls.2024.1466279

**Published:** 2024-08-22

**Authors:** Tania Ho-Plágaro, Muhammad Usman, Janne Swinnen, Joske Ruytinx, Françoise Gosti, Isabelle Gaillard, Sabine D. Zimmermann

**Affiliations:** ^1^ IPSiM, Univ Montpellier, CNRS, INRAE, Institut Agro, Montpellier, France; ^2^ Research Groups Microbiology and Plant Genetics, Department of Bioengineering Science, Vrije Universiteit Brussel, Brussel, Belgium

**Keywords:** ectomycorrhizal symbiosis, cation diffusion facilitator (CDF), *Hebeloma cylindrosporum*, mycorrhizal gene activation, *Pinus pinaster*, yeast complementation, Zn transporter

## Abstract

Zinc (Zn) shortage is a common micronutrient deficiency affecting plants worldwide, while Zn toxicity may occur when this metal is in excess. Ectomycorrhizal (ECM) fungi are known to be able to modulate the transfer of macro- and microelements, among them Zn, to the plant. However, the underlying mechanisms are not well understood. We identified the *HcZnT2* gene from the ECM fungus *Hebeloma cylindrosporum*, encoding a member of the Cation Diffusion Facilitator (CDF) family including Zn transporters, and analyzed its transcriptional regulation, the transport function by yeast complementation experiments, and its subcellular localization using a GFP fusion protein in yeast. *HcZnT2* is highly induced during mycorrhization of *Pinus pinaster*, and upregulated in presence of the host plant root even without any direct contact. However, *HcZnT2* is repressed by Zn excess conditions. By functional expression in yeast, our results strongly support the ability of HcZnT2 to transport Zn and, to a lesser extent, manganese. HcZnT2 localization was associated with the endoplasmic reticulum of yeast. Mycorrhizal gene activation at low external Zn suggests that the Zn transporter HcZnT2 might be important for the early establishment of the ECM symbiosis during Zn deficiency, rather than under Zn excess. HcZnT2 arises as an extremely remarkable candidate playing a key role in Zn homeostasis and regulation in ectomycorrhiza.

## 1 Introduction

Mycorrhizas are widespread mutualistic associations between fungi and plant roots, that are thought to have played a key evolutionary role in facilitating the colonization of the terrestrial ecosystems by plants, around 450 million years ago (Rich et al., 2021). Among the diverse advantages arising from the mycorrhizal symbiosis, the most outstanding one is the nutritional benefit obtained by both interacting partners, in which mycorrhizal fungi provide mineral nutrients and water to their host plants in exchange for sugar and lipids (Marschner and Dell, 1994; Solaiman and Saito, 1997; Read and Perez-Moreno, 2003; Keymer et al., 2017). However, the regulation of these exchanges is still poorly described (Carbonnel and Gutjahr, 2014; Garcia et al., 2016).

It is well known that mycorrhizal fungi improve the acquisition of minerals such as phosphorous, nitrogen and potassium from the soil, and are able to transfer these macronutrients to the plant, improving their nutritional status (Garcia et al., 2016). Although less studied, several reports also point to the ability of mycorrhizal symbiosis to optimize plant micronutrition (Ruytinx et al., 2020). Several micronutrients have the particularity to be essential for fungal and plant growth but they can become toxic at high concentrations. In this context, mycorrhizal fungi are not only able to promote micronutrient transfer to the plant under deficiency conditions, but also to restrict this flux if these minerals are present at toxic levels (Adriaensen et al., 2004). This is the case of zinc (Zn), whose deficiency is the most widespread and recurrent in pasture and crop plants worldwide (Alloway, 2008), while in some cases Zn toxicity conditions have been also reported to occur and to have negative effects on plants, generally in areas affected by mining or metallurgic activities (Ernst, 1990; Alloway, 2008; Nagajyoti et al., 2010). However, the regulation of Zn transport along the soil-fungi-plant continuum and the mechanisms involved are still unclear.

Fungal Zn transporters have mainly been identified in two protein families: the CDF (Cation Diffusion Facilitator) and the ZIP (Zrt/Irt-like protein) transporter families (Eide, 2006). Both families also include iron (Fe) and manganese (Mn) transporters, and several members in these families have been demonstrated to be able to transport in addition other divalent metal ions, such as cadmium (Cd), cobalt (Co), and nickel (Guerinot, 2000; Montanini et al., 2007). ZIP transporters have been reported to transport extracellular or stored Zn into the cytoplasm (Kambe et al., 2006), while CDFs transport Zn into organelles or out of the cell, decreasing the cytoplasmic Zn levels (Montanini et al., 2007). Several CDFs have been identified and described in ectomycorrhizal (ECM) fungi forming symbioses with woody plants. In *Hebeloma cylindrosporum*, HcZnT1 mediates Zn storage in endoplasmic reticulum (ER)-derived vesicles (Blaudez and Chalot, 2011). SlZnT1 and RaCDF1 direct vacuolar Zn storage in *Suillus luteus* (Ruytinx et al., 2017) and in *Russula atropurpurea* (Sácký et al., 2016), respectively. RaCDF2 exports Zn in *R. atropurpurea* (Sácký et al., 2016). Finally, RaZIP1 and SlZRT1 are also involved in Zn uptake in *R. atropurpurea* (Leonhardt et al., 2018) and *S. luteus* (Ruytinx et al., 2017), respectively.

Interestingly, an RNA-seq analysis performed with the ectomycorrhizal fungus *H. cylindrosporum* associated with young pine roots compared to free fungal mycelia (Doré et al., 2015) revealed that a gene encoding a putative fungal CDF member (jgi protein ID 421984) was the one showing the highest induction within the complete fungal transportome (Guerrero-Galán, 2017). Phylogenetically, this gene was closely related to the *HcZnT1* gene characterized previously as a Zn transporter involved in detoxification by Blaudez and Chalot (2011). In the present study, we characterized the function and localization of this new putative Zn transporter, here named HcZnT2, by heterologous expression in different yeast strains including *Saccharomyces cerevisiae* wild-type (WT) or Zn-, Mn- and Cd-hypersensitive mutant strains. Moreover, we analyzed the transcriptional regulation of *HcZnT2* by the host plant *Pinus pinaster* and by external Zn concentrations.

## 2 Materials and methods

### 2.1 Plant and fungal material and culture conditions

The homokaryotic strain h7 of the ECM basidiomycete *H. cylindrosporum* Romagnesi (Debaud and Gay, 1987) and maritime pine seeds (*P. pinaster* Soland in Ait. from Medoc, Landes-Sore-VG source, France) were used for the experiments.

To analyze the tolerance of *H. cylindrosporum* to different external Zn concentrations, actively growing fungal implants (diameter of 8 mm) were grown on solid (12 g l^-1^ agar-agar) N6 medium (N6: Boukcim and Plassard (2003) with vitamines by Morizet and Mingeau (1976); *Cf*. **Supplementary Material**) at different ZnSO_4_ concentrations (0, 0.30, 3, 30, 310 and 3100 μM). After two weeks of incubation at 26°C, the radial growth and dry weight of the fungus were measured.

In order to simulate the space of the Hartig net, a symbiotic interface-mimicking experiment was performed at different Zn concentrations (0, 30, 1000 µM ZnSO_4_), based on the protocol described (Becquer et al., 2017; Torres-Aquino et al., 2017). Fungal mycelia were used for RNA extraction and RT-qPCR analysis (**Supplementary Material**). Data analyses for gene expression levels were performed as described by Cuéllar et al. (2010) and Guerrero-Galán et al. (2018).

### 2.2 *HcZnT2* cloning, plasmids and yeast strains

A cDNA library of *H. cylindrosporum* mycelia (collected from the symbiotic-interface mimicking assay) was used to amplify the *HcZnT2* full-length cDNA sequence and to obtain the pYES2::*HcZnT2* construct for functional expression and the pYES::*HcZnT2*-*EGFP* construct (Hamilton et al., 1987) for localization in yeast (**Supplementary Material**).

For heterologous functional expression and subcellular localization in yeast (Gietz and Woods, 2006), the *S. cerevisiae* WT strain BY4741 and three derived mutants were used: the Zn-hypersensitive double mutant strain *Δzrc1 Δcot1*, the Mn-hypersensitive mutant *Δpmr1*, and the Cd-hypersensitive mutant *Δycf1* (*Cf*. **Supplementary Material**). Double transformation with the pYX222 (his+) empty vector was done to avoid addition of the Zn chelator histidine (Krämer et al., 1996; Murphy et al., 2011).

### 2.3 Functional complementation of yeast and subcellular localization

For metal tolerance assays, transformed yeasts were grown to mid log phase (OD_600_ = 1) in liquid SD-His-Ura medium with 2% w/v D-galactose instead of D-glucose (induction medium). Yeast cells were pelleted, washed with sterile distilled water, and adjusted to OD_600_ = 1. A 1/10 dilution series was prepared (10^0^, 10^-1^, 10^-2^, and 10^-3^). Drop assays were performed for three independent yeast colonies on SD-His-Ura control induction medium and on SD-His-Ura induction medium supplemented with different concentrations of ZnSO_4_, MnSO_4_ or CdSO_4_.

For subcellular localization of HcZnT2-EGFP fusion protein (**Supplementary Material**), yeast transformants were grown to mid-log phase OD_600_ = 1 on SD-His-Ura induction medium, followed by a 40 min incubation at 30°C in the dark in the presence of the corresponding dye in order to stain the vacuolar membrane or the nuclei. Staining of the vacuolar membrane was performed based on the protocol described by Vida and Emr (1995), by incubating the cells in 40 µM FM4-64 (Molecular Probes, Invitrogen, Carlsbad, CA, United States) SD-His-Ura induction medium. For nuclei visualization, yeasts were incubated in SD-His-Ura induction media containing 10 μg ml^–1^ of the cell-permeant nuclear counterstain Hoechst 33342 (Invitrogen, Carlsbad, CA, United States).

### 2.4 Bioinformatics analysis and construction of a phylogenetic tree of CDF family proteins

Amino acid sequence alignment and calculation of amino acid sequence identity between HcZnT2 and the close homologue HcZnT1 were performed with Clustal Omega (Sievers et al., 2011).

The structural model of HcZnT2 was calculated using the AlphaFold2 advanced notebook on Google Colab (Jumper et al., 2021). The default settings were applied, specifically utilizing *de novo* generation of multisequence alignments with mmseqs2. We generated five models for each prediction with 1 ensemble, 12 recycles, and 1 random seed.

A phylogenetic tree was constructed with CDF family proteins (Montanini et al., 2007) of *H. cylindrosporum* and selected fungal species (*Cf*. **Supplementary Material**).

## 3 Results

### 3.1 *HcZnT2* encodes a putative zinc transporter

A cDNA library of *H. cylindrosporum* mycelia was used and specific primers were designed in order to amplify the full-length cDNA encoding the protein corresponding to the CDF-type gene jgi ID 421984. The amplified PCR product was ligated into the pYES2 vector. Three independent clones were sequenced and shown to be identical (GenBank OR167112). However, with respect to the sequence annotated in the Mycocosm database (https://mycocosm.jgi.doe.gov/cgi-bin/dispTranscript?db=Hebcy2&id=421984&useCoords=1), the sequenced cDNA showed a shorter length and slight nucleotide differences probably due to some intron splicing errors of the initial annotation. The size of the amplified fragment was 1095 bp length (in contrast to the 1116 bp sequence from the database). The resulting encoded amino acid sequence contains 364 aa.

**Figure 1:**
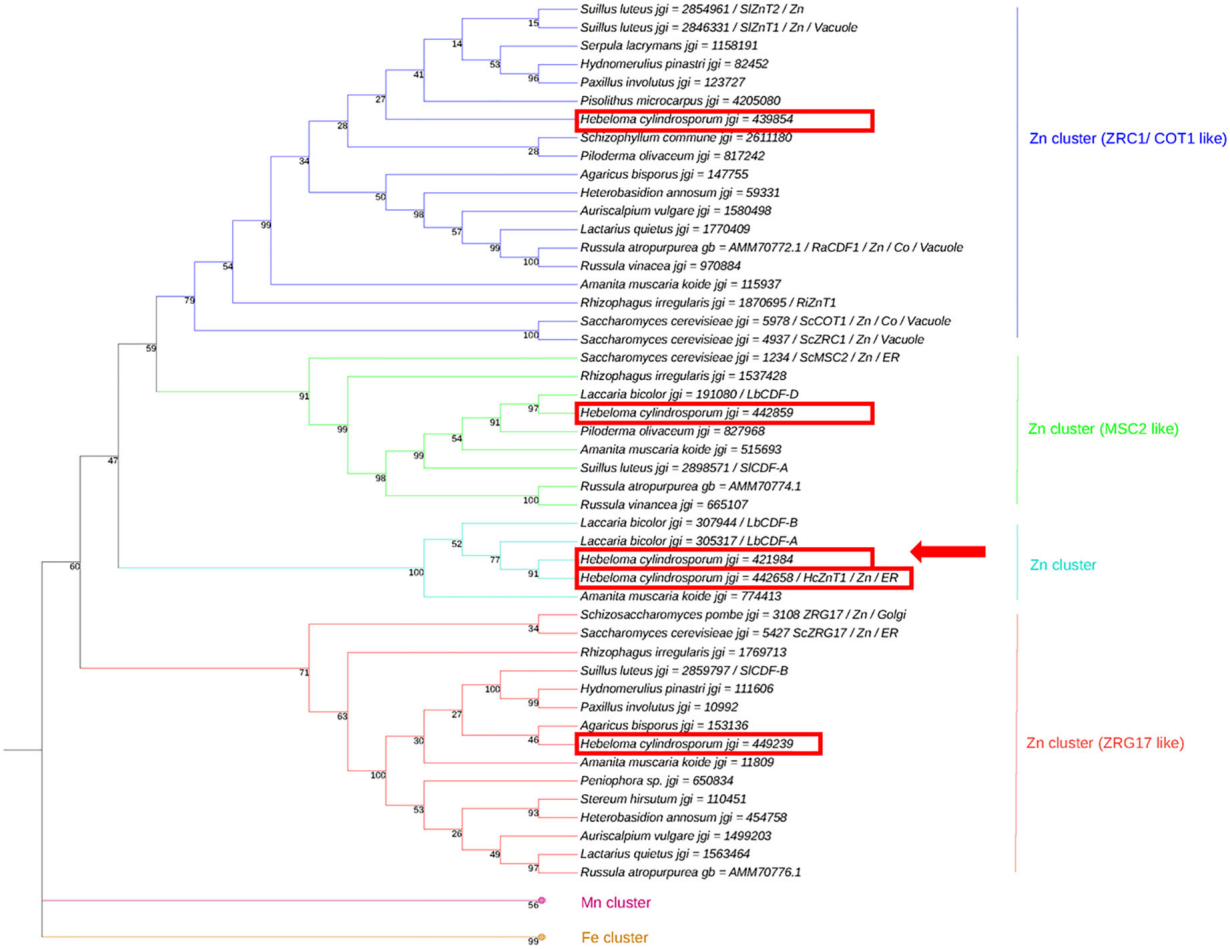
Phylogenetic analysis of fungal proteins from the cation diffusion facilitator (CDF) family. Neighbor-Joining (NJ) tree of putative CDF proteins from *Saccharomyces cerevisiae*, *Hebeloma cylindrosporum* and other fungal species. Bootstrap values (1000 replicates) are indicated and branch lengths are proportional to phylogenetic distances. Localization and substrate (metal) are indicated for functionally characterized proteins. Mn and Fe clusters are collapsed and used as outgroups. *H. cylindrosporum* sequences are framed, and HcZnT2 is indicated by an arrow.

Phylogenetic analysis predicted jgi ID 421984 to be located within the cluster of ER/Golgi localized Zn transporter (**Figure 1**). Proteins assembled in three major clusters according to the transported metal substrate: Zn, Mn or Fe. The protein corresponding to jgi ID 421984 resided within the Zn cluster. Within this major Zn sub-group, four clusters were identified. Three of these clusters contain previously well-characterized *S. cerevisiae* CDF transporters that were named accordingly, ZRC1/COT1, MSC2 and ZRG17 (Kamizono et al., 1989; Conklin et al., 1992; Li and Kaplan, 2001; Ellis et al., 2005). The fourth cluster fell in between the ZRG17-like and MSC2-like cluster of ER/Golgi-localized transporter proteins and includes jgi ID 421984 along with the previously characterized HcZnT1. Despite the fact that bootstrap values were relatively low (47 – 59) and more sequences would be needed to be included to better support and resolve evolution of proteins within these particular clusters, an ER/Golgi localization can likely be assumed for members of the fourth cluster based on its placement within the overall tree. Both *H. cylindrosporum* proteins were very closely related, sharing 80.66% amino acid sequence identity. For this reason, the protein corresponding to the jgi ID 421984 was named systematically HcZnT2.

In order to predict the structure of HcZnT2, we performed a protein modelling using AlphaFold2. The representative structure indicated that the HcZnT2 protein possess six putative transmembrane helix domains (**Supplementary Figure S1**), as it is experimentally demonstrated for bacterial members (Anton et al., 1999; Wei and Fu, 2005).

### 3.2 *HcZnT2* expression is dependent on the host plant and on zinc concentrations

Taking advantage from transcriptomic data of *H. cylindrosporum* in mycorrhizal association with maritime pine, we analysed the expression regulation of the fungal genes coding for membrane transport systems. Precisely, a RNA-seq analysis was carried out by Doré et al. (2015) with the ECM fungus *H. cylindrosporum* (WT dikaryotic strain TV98 IV3) from ECM pine root tips under two environmental conditions, *in vitro* (in same culture conditions) and in soil (mycorrhizal roots in soil). For *in vitro* transcript analysis, fungal gene expression from ECM root tips synthesized by 3-week-old axenic cultures using *P. pinaster* as host was compared to six-day-old *H. cylindrosporum* free-living mycelia on the same controlled medium (GSE63868 GEO dataset). For the soil transcriptome, ECM root tips were produced in the greenhouse by inoculation of 20-day-old pine seedlings with the fungus and following 6-month co-cultivation on a different substrate (80% organic matter) (GSE66156 GEO dataset). Based on the raw transcriptomic data generated by Doré et al. (2015), we observed that the *HcZnT2* gene expression was 292-fold induced in the *in vitro* ECM roots compared to the fungus alone and, strikingly, Guerrero-Galán (2017) found that in this experiment *HcZnT2* was the most highly induced transporter-encoding gene in the ECM roots, among all the identified genes encoding fungal membrane transport systems. Moreover, data analyses performed by Guerrero-Galán (2017) confirmed that this mycorrhiza-induced gene expression also occurred in the soil transcriptome, though to a lesser extent with a 34-fold induction, explained by divergent culture and nutritional conditions. In addition, another RNA-seq study carried out at early stages of *H. cylindrosporum* - *P. pinaster* interactions (GSE93184 GEO dataset; Doré et al., 2017) showed that *HcZnT2* was already 30-fold induced at the initial formation of the fungal mantle, when hyphal patches attached to host epidermal root cells start to appear (4 days post-inoculation). However, *HcZnT2* was not significantly induced during rhizosphere colonization at 2 days post inoculation (Doré et al., 2017), when hyphae are not yet adhered to the root tissues.

**Figure 2:**
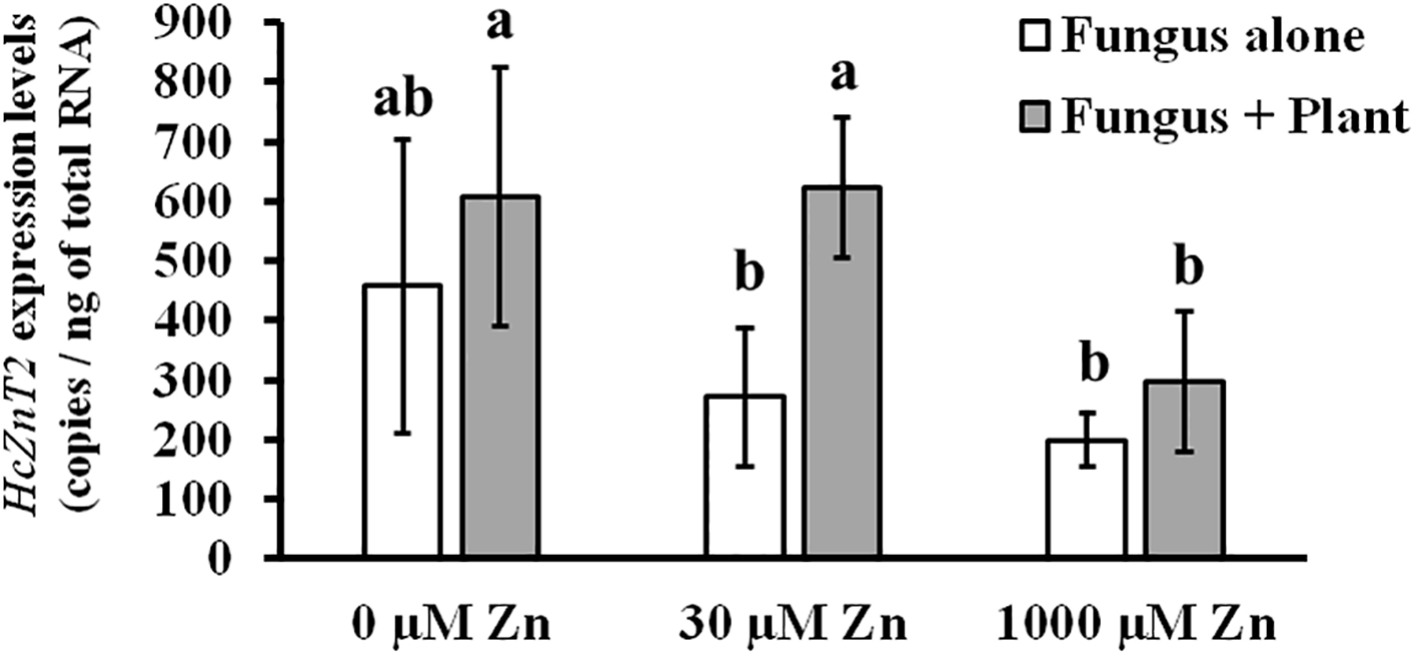
*HcZnT2* gene expression in a symbiotic interface-mimicking experiment at different zinc concentrations. Determination of transcript levels of *HcZnT2* in *Hebeloma cylindrospourm* cultures after 48 h incubation in induction media in the absence or in the presence of pine seedlings at different Zn concentrations (0 µM, 30 µM and 1000 µM). Values correspond to mean ± SE (n=4). Bars with a same letter are not significantly different (P>0.05) according to Tukey´s multiple comparison test.

Regarding this observed significant transcriptional upregulation, we wondered if *HcZnT2* gene induction in ECM roots requires the physical contact between plant and fungus, meaning established symbiosis, or only a kind of signaling by the presence of the host plant roots. In addition, data were needed to know if *HcZnT2* transcriptional regulation might be dependent on Zn concentrations in the external media. For this purpose, a short-term symbiotic interface-mimicking experiment (Becquer et al., 2017) was performed with fungal cultures in the presence or absence of young pine roots in the same medium but without direct contact at different Zn concentrations (0, 30 and 1000 µM). The 30 and 1000 µM Zn concentrations were selected as optimal and toxic Zn concentrations, respectively, based on Zn tolerance tests for fungal growth (**Supplementary Figure S2**). First, we observed a reduction of *HcZnT2* gene expression with higher Zn concentrations (**Figure 2**), indicating rather a role under Zn deficiency than in detoxification conditions. Excitingly, at least at 30 µM external Zn, *HcZnT2* gene expression was significantly upregulated by the presence of pine seedlings after 48 h of mycelia incubation (**Figure 2**), suggesting the involvement of HcZnT2 in early plant-fungal signaling.

### 3.3 HcZnT2 is able to restore the growth of Zn- and Mn- hypersensitive yeast mutants exposed to metals

**Figure 3:**
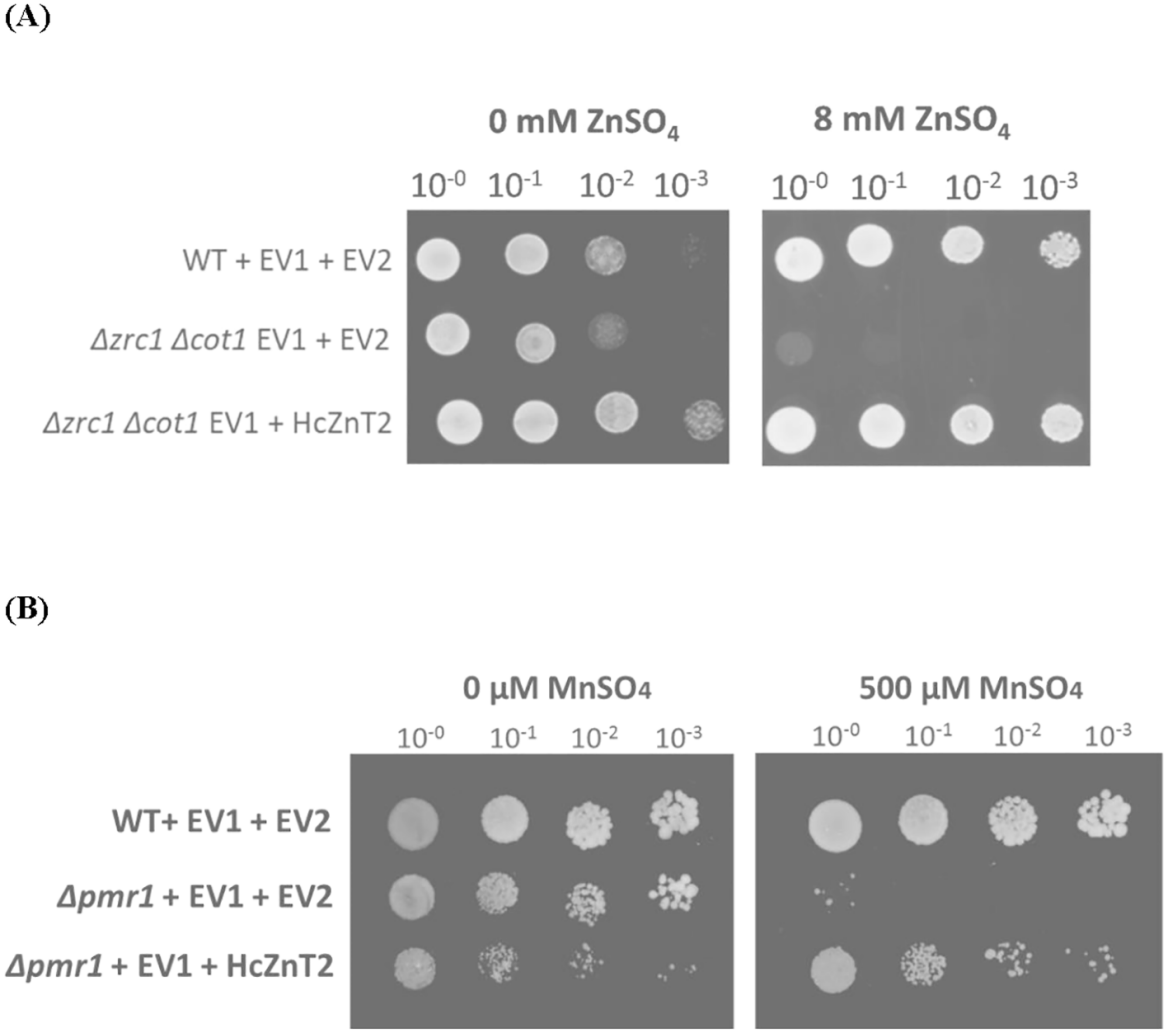
HcZnT2 restores transporter deficient yeast mutants suggesting Zn and Mn transport. Cultures with an OD_600 =_ 1 were 10-fold serial diluted (10^0^, 10^-1^, 10^-2^, and 10^-3^), as indicated above the panels. 10-µl-drops of these serial dilutions were spotted on SD-His-Ura induction control medium (first column) or supplemented (second column). **(A)** Functional complementation of the zinc-hypersensitivity of the *Δzrc1 Δcot1* yeast mutant by HcZnT2. Wild-type BY4741 (WT) and double mutant *Δzrc1 Δcot1* yeast strains harbouring the empty vector pYX222 (EV1) that brought histidine autotrophy and either pYES2 empty vector (EV2) or pYES2::HcZnT2 were used. Medium was supplemented with 8 mM ZnSO_4_ (second column). Pictures were taken after 2 days of growth for the control (0 mM Zn) and after 4 days for the Zn treatment. One representative example out of three independent experiments is shown. **(B)** Functional complementation of the manganese-hypersensitivity of the *Δpmr1* yeast mutant by HcZnT2. Wild-type BY4741 (WT) and mutant *Δpmr1* yeast strains harbouring the empty vector pYX222 (EV1) that brought histidine autotrophy and either pYES2 empty vector (EV2) or pYES2::HcZnT2 were used. Medium was supplemented with 500 µM MnSO_4_ (second column). Pictures were taken after 6 days of growth. One representative example out of three independent experiments is shown.

*HcZnT2* cDNA was heterologously expressed in the *S. cerevisiae Δzrc1 Δcot1* double mutant strain, which is defective in vacuolar Zn transport and then unable to grow on high concentrations of Zn because of the lack of detoxification of the cytosol (MacDiarmid et al., 2000). A drop test was performed and yeast growth was monitored on either SD-His-Ura induction control media (0 mM ZnSO_4_) or in the same media supplemented with 8 mM ZnSO_4_. While transformation with the empty vectors did not complement at all the Zn hypersensitive phenotype of the *Δzrc1 Δcot1* strain, HcZnT2 showed the ability to completely restore yeast growth of this mutant at 8 mM ZnSO_4_ (**Figure 3A**).

*HcZnT2* was also expressed in *S. cerevisiae* yeast mutants lacking the endogenous metal transporters for Mn (*Δpmr1*) and Cd (*Δycf1*), being unable to grow on Mn and Cd, respectively (Li et al., 1997; Ton et al., 2002). The results showed that the defective growth of the *Δpmr1* strain at 500 µM MnSO_4_ was partially restored after transformation with *HcZnT2* (**Figure 3B**). However, *HcZnT2* does not confer Cd tolerance to the Cd-hypersensitive *Δycf1* mutant at any of the tested concentrations (**Supplementary Figure S3**). These results suggest that HcZnT2 transports rather specifically Zn and, in a lesser extent Mn, but not Cd.

### 3.4 HcZnT2 has an ER subcellular localization in yeast

**Figure 4:**
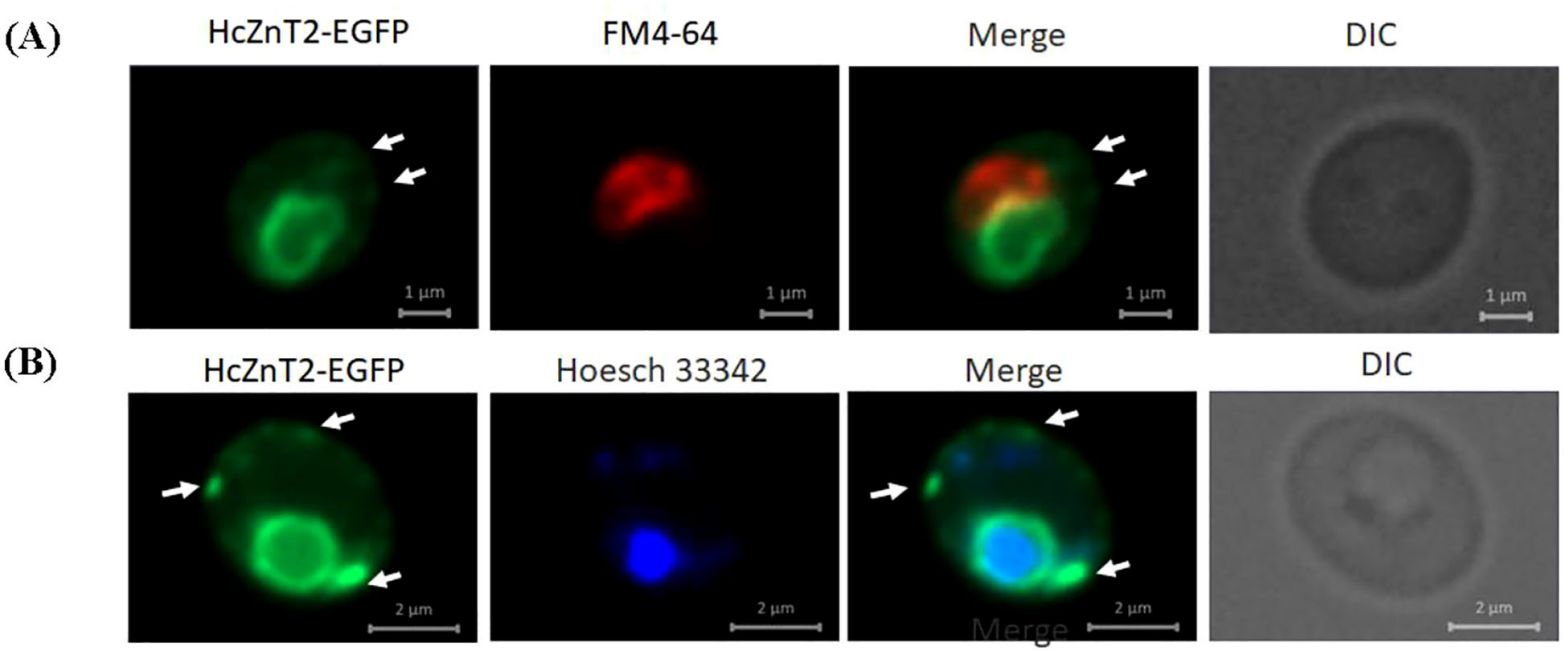
HcZnT2-EGFP is likely localized in the endoplasmic reticulum in yeasts. WT BY4741 yeast cells expressing the HcZnT2-EGFP fusion protein were grown in induction media and incubated with the red fluorescent dye FM4-64 at 30°C to label the vacuolar membranes **(A)** or with the blue fluorescent dye Hoechst 33342 at 30°C to label the nuclei **(B)**. The corresponding merged images (EGFP + FM4-64 or EGFP + Hoescht 33342) and DIC (differential interference contrast) images are shown. White arrows indicate EGFP-labelled cytoplasmic strands, which probably correspond to ER extensions connecting the nuclear envelope to the peripheral ER structures.

The HcZnT2 transporter was predicted to be ER/Golgi-associated by the phylogenetic analysis (**Figure 1**). To test the subcellular localization of the fungal HcZnT2 transporter, a heterologous approach was pursued by expressing a functionally active HcZnT2-EGFP fusion protein in yeast. In cells counter-stained with the lipophilic red fluorescent vacuolar dye FM4-64 (Vida and Emr, 1995), we observed that the EGFP fluorescence was forming a ring-like pattern that did not co-localized with the FM4-64 labelled vacuoles (**Figure 4A**). However, nuclei staining performed with the blue fluorescent dye Hoechst 33342 revealed that the EGFP ring was surrounding the nucleus (**Figure 4B**), what is typical of ER structures forming the nuclear envelope. In fact, EGFP fluorescence was also found as strands and in the cell periphery (arrows in **Figure 4**), what is characteristic of the outward ER extensions and of the peripheral ER, respectively.

## 4 Discussion

ECM fungi are known by their capability to improve plant mineral nutrition, but also to alleviate the negative effect caused by toxic trace metals on plants. In the case of plant micronutrients, which are essential for plants but toxic when present in excess, ECM fungi are reported to play a dual role by either promoting or avoiding plant acquisition of these trace elements depending on their concentrations in the soil (Denny and Wilkins, 1987; Bücking and Heyser, 1994). However, little information is known about the mechanisms involved.

In this study, we identified the *HcZnT2* fungal gene from *H. cylindrosporum* highly induced in ECM pine roots, which encodes a member of the CDF family falling into the Zn transporter cluster. In particular, the HcZnT2 protein is phylogenetically close to the Msc2-like and Zrg17-like proteins (**Figure 1**). Some fungal proteins belonging to the Zn transporting group have been already characterized, and their ability to transport Zn has been confirmed. This is the case of the *S. cerevisiae* Zrc1 and Cot1 (MacDiarmid et al., 2000), the *R. atropurpurea* RaCDF1 (Sácký et al., 2016), the *S. luteus* SlZnT1 (Ruytinx et al., 2017), and the highly related *H. cylindrosporum* HcZnT1 transporter (Blaudez and Chalot, 2011).

Here, the ability of HcZnT2 to transport Zn was suggested by complementation tests using a yeast double mutant deficient in vacuolar Zn transport. As predicted by the phylogenetic tree (**Figure 1**), HcZnT2-GFP revealed a pattern typical of ER targeting, with fluorescence mainly observed surrounding the nucleus and also distinguishable as structures in the cell periphery, and as cytoplasmic strands connecting both the nucleus and periphery (**Figure 4**). Although ER-specific labelling would be required to confirm further the ER localization, our results strongly indicate that the fungal HcZnT2 transporter is located and might allow the storage of Zn in the ER (or in some nearby vesicles or compartment/s), being able to restore the growth of yeast mutants impaired in Zn storage in the vacuole upon Zn toxicity conditions. Similar results have been obtained for the close homologue HcZnT1 protein, which shares 80.66% amino acid sequence identity with HcZnT2. An ER fluorescence pattern localization has been also observed for HcZnT1::GFP when expressed in yeast, and the Zn-sensitive phenotype of both *cot1* and *zrc1* yeast mutants is fully complemented by HcZnT1 (Blaudez and Chalot, 2011).

Depending on the fungal species, Zn acquisition or storage has been shown to occur in different compartments. *S. cerevisiae* is able to transport Zn into the ER to assure proper functioning of ER (Ellis et al., 2004). For *S. luteus* and *Suillus bovinus*, an accumulation of Zn in the vacuole has been reported (Ruytinx et al., 2013, 2017). The arbuscular mycorrhizal (AM) fungus *Glomus intraradices* (Gonzalez-Guerrero et al., 2008) and likely the ECM fungus *Paxillus involutus* (Tuszynska et al., 2006) are also able to store Zn in the vacuoles. So far, it is not known if *H. cylindrosporum* has the ability to accumulate Zn in the ER. In *H. cylindroporum* mycelia, through zinquin labelling, Blaudez and Chalot (2011) only observed accumulations of Zn in numerous small punctuate vesicles which did not coincide with vacuoles and resembled the mammalian and *Schizosaccharomyces cerevisiae* cytoplasmic vesicles storing Zn, commonly known as zincosomes (Li and Kaplan, 2001; Devirgiliis et al., 2004; Wellenreuther et al., 2009). However, the storage of Zn in other possible fungal compartments cannot be discarded, as zinquin binding to Zn^2+^ is impaired by the acidic pH present in some compartments (including the vacuoles), and maybe also by the presence of Zn-ligand complexes, as suggested by Blaudez and Chalot (2011). Although it is tempting to speculate that HcZnT2 (and HcZnT1) from *H. cylindrosporum* allows storage of Zn in the ER or some nearby compartments, the correspondence of these structures with the so-called zincosomes remains to be elucidated.

The high similarity between the transporters HcZnT1 and HcZnT2, together with their common ability to transport Zn and to be localized in or next to the ER, points to a functional redundancy of both transporters concerning Zn homeostasis regulation. However, both genes are clearly regulated differently as only *HcZnT2* shows this significant and pronounced mycorrhiza-dependent transcriptional regulation. Further *HcZnT2* expression analyses in isolated fungal cultures grown at different Zn concentrations, and parallel *HcZnT1* expression data in such experiments, could help to elucidate differences on the role of HcZnT1 and HcZnT2 in Zn homeostasis regulation. Both transporters may also differ in their specificity to transport other alternative metals, as some CDF Zn transporters have been reported to transport other metals in addition to Zn. For example, the yeast Zn transporters ScCot1 and ScZrc1 are also able to transport Co or Cd, respectively (Kamizono et al., 1989; Conklin et al., 1992). With this respect, we confirmed that the HcZnT2 transporter has some ability to transport Mn, while HcZnT1 is probably not able to transport this metal (Blaudez and Chalot, 2011).

Interestingly, data from previous RNA-seq analyses (Doré et al., 2015, 2017) showed that *HcZnT2* is induced from the initial formation of the fungal mantle (30-fold) to developed ECM roots, in both soil (34-fold) and *in vitro* (292-fold) conditions. This important mycorrhiza-induced expression level increase of *HcZnT2* was found to be the highest among all identified membrane transport systems from the ECM fungus (Guerrero-Galán, 2017). Here, a short-term symbiotic interface-mimicking experiment was performed at different Zn concentrations (0, 30 and 1000 µM). *HcZnT2* was repressed at 1000 µM Zn concentration (toxic Zn levels) indicating a role of HcZnT2 under Zn deficiency or low Zn concentrations, rather than in Zn detoxification conditions. Excitingly, a high transcriptional induction of *HcZnT2* was found by the presence of the host plant roots after only 48 h of co-incubation without physical contact at 30 µM external Zn, suggesting that *HcZnT2* is induced by early signaling between the symbiotic partners when external Zn levels are optimal for fungal growth. This remarkable transcriptional induction of *HcZnT2* by the presence of host plant roots confirmed not only the mycorrhiza-induced expression from the former RNA-seq data but goes beyond that indicating the involvement of *HcZnT2* in early signaling between the symbiotic partners or as target of a priming response, preparing the fungus for symbiotic transport processes. It is worth mentioning that the Zn levels considered here as “optimal” are based on Zn tolerance tests performed with fungal axenic cultures (or liquid co-cultures), so the optimal levels for plants in soil might be different. Actually, optimal Zn in soils for improved plant yields is reported to be in a range of 4-10 ppm in agricultural fields (Pande et al., 2007; Hussain et al., 2013; Liu et al., 2017), and Zn content reported in forests soils ranges from 20 to 300 ppm (Zhao et al., 2021). These soil Zn contents are far above the 30 µM (equivalent to 0.2 ppm) Zn used here in liquid cultures. Then, 30 µM Zn might correspond to a Zn deficient treatment considering the plant needs. This would explain the similar results obtained for the expression of *HcZnT2* in both 0 and 30 µM Zn treatments, thus supporting the idea of an induction of *HcZnT2* by the presence of the host plant by early signaling when the plant is under Zn deficient conditions, as a potential mechanism to cope with this nutritional stress. Zn sequestration was shown for *Hebeloma mesophaeum*, a Zn-accumulating ectomycorrhizal (EM) species frequently associated with metal disturbed sites (Sácký et al., 2014). The role of ECM fungi in modulating Zn uptake by the host plant was shown also for poplar in metalliferous soil (Langer et al., 2012), Moreover, Blaudez and Chalot (2011) suggested a dual role for HcZnT1 in Zn homeostasis of *H. cylindrosporum* for detoxification of the cytosol and supply of Zn to the ER.

Regarding the role of Zn within early steps of cross-talk between plant host and fungal symbiont and during symbiosis establishment, interesting results obtained in yeast point to the implication of Zn homeostasis in Golgi membrane trafficking. In fact, a CDF transporter, Cis4 in fission yeast *Schizosaccharomyces pombe* was found to mediate Zn uptake in the cis-Golgi (Fang et al., 2008; Jaiseng et al., 2012). Mutant phenotypes, as weak cell wall and decreased phosphatase secretion, indicated impaired membrane trafficking. Even though yeast is far away from ECM association, these findings would fit with a role of Zn regulation in the steps of highly active membrane trafficking during the symbiosis establishment, as mycorrhiza formation requires many membrane dynamics (Leborgne-Castel and Bouhidel, 2014). In line with the hypothesis that Zn homeostasis might play an essential role in mycorrhizal symbiosis, in AM symbiosis in *Medicago truncatula*, another type of Zn transporter, MtZIP14, was recently found to be involved in fungal colonization (Watts-Williams et al., 2023). In general, AM symbiosis is thought to enhance plant Zn uptake (Lehmann et al., 2014). Mycorrhizal networks of AM fungi and expression changes of Zn transporters in fungi and plants might be involved (Cardini et al., 2021). Moreover, a tight interaction between Zn and P pathways in AM symbiosis is suggested in maize (Yu et al., 2024). Further studies will be needed to analyze if Zn content in the roots is affected by *H. cylindrosporum* colonization, and to dissect more in detail the biological function of the fungal HcZnT2 during the ECM symbiotic interaction in different environmental and nutritional conditions.

Altogether, the early and significant mycorrhiza-dependent transcriptional regulation of *HcZnT2* indicates clearly the importance of the control of Zn transport and homeostasis in the establishment and functioning of the ECM association with pine roots, probably linked to membrane dynamics. As the most highly mycorrhiza-induced membrane transport system in *H. cylindrosporum*, HcZnT2 constitutes a strong candidate as key player in ECM symbiosis.

## Data Availability

The datasets presented in this study can be found in online repositories. The names of the repository/repositories and accession number(s) can be found in the article and in **Supplementary Material**.
