# Supplementary material for: HcZnT2 is a highly mycorrhiza-induced zinc transporter from Hebeloma cylindrosporum in association with pine
Source: Front Plant Sci. 2024 Aug 22;15:1466279. doi: 10.3389/fpls.2024.1466279 (PMC11374630; doi:10.3389/fpls.2024.1466279)
Supplement: Supplementary file 1 [file DataSheet1.pdf]

Supplementary Material

1 Supplementary Materials and Methods

1.1 Fungal cultures

For fungal cultures, the N6 medium was prepared as described by Boukcim and Plassard (2003): 6 mM KNO_3, 3 mM NaH_2PO_4, 1 mM $MgSO_4 \cdot 7H_2O$, 4 mM KCl, 0.5 mM $CaCl_2$, 0.04 mM Fe (III) citrate, 0.3 µM thiamine-HCl, 0.2 $ml \cdot l^{-1}$ Morizet and Mingeau (1976) microelements solution (3.08 $g \cdot l^{-1}$ $MnSO_4 \cdot H_2O$, 4.41 $g \cdot l^{-1}$ $ZnSO_4 \cdot 7H_2O$, 2.82 $g \cdot l^{-1}$ H_3BO_3, 0.98 $g \cdot l^{-1}$ $CuSO_4 \cdot 5H_2O$, 0.29 $g \cdot l^{-1}$ $Na_2MoO_4 \cdot 2H_2O$), and 5 $g \cdot l^{-1}$ glucose, pH 5.5.

In order to simulate the space of the Hartig net, a symbiotic interface-mimicking experiment was performed based on the protocol described by Becquer et al. (2017) and Torres- Aquino et al. (2017). Briefly, three 2-month-old pine seedlings grown in liquid N1 medium and one 3-week-old fungal mycelium (grown in liquid N6 media) were incubated together but without physical contact for up to 48 h in interaction medium (IM) without P and C source ($MgSO_4$ 0.2 mM; $CaCl_2$ 0.5 mM; MES at 5 mM; TRIS 5 mM; at pH 5.5) at different Zn concentrations (0, 30, 1000 µM $ZnSO_4$). At the end of the experiment, fungal mycelia were stored at -80ºC for further RNA extraction.

1.2 Phylogenetic tree

Sequences of functionally characterized CDF family transporters of the model fungus *S. cerevisiae* (Table **S1**) were extracted from the Uniprot Knowledgebase (UniprotKB) and used as a query to retrieve CDF family proteins of *H. cylindrosporum* and selected fungal species (Table **S2**). BLASTp and BLASTx searches were run at the respective genome portals at MycoCosm of the Joint Genome Institute. The retrieved sequences were imported in the CLC Main Workbench (Qiagen, France) for analysis. They were considered CDF family proteins and included in further analysis when 350-700 amino acids long, having 6 predicted transmembrane domains and showing a CDF conserved domain or signature sequence (Montanini et al., 2007). The identified CDF family proteins were aligned along with previously characterized fungal CDF family transporters (retrieved from UniprotKB or NCBI protein database, Table **S2**) using a progressive alignment algorithm and default settings. A phylogenetic tree was constructed using the Neighbor-Joining (NJ) method with Jukes-Cantor protein distance measure to infer evolution and predict function of the identified transporter proteins. Bootstrap were included using 1000 replicates. The constructed tree was exported from the CLC main workbench in Newick format and annotated in the online tool Interactive Tree of Life (iTO; https://itol.embl.de/).

1.3 RNA extraction and RT-qPCR analysis

Fungal mycelia were used for RNA extraction with the Spectrum™ Plant Total RNA Kit (Sigma). RNAs were treated with DNAse (deoxyribonuclease I, amplification grade, Invitrogen), and cDNA synthesis was performed by retrotranscription using SuperScript™ IV Reverse Transcriptase (Invitrogen). Real-time PCRs were performed in the LightCycler® 480 Real-Time system (Roche, Mannheim, Germany) using a reaction mix containing 10 ng cDNA, 5 µl SYBR® Premix Ex Taq ™ II Tli RNaseH Plus (TaKaRa Bio Inc., Dalian, China) and 0.5 µM of each forward (5’-CATTAAATAACATCGGAGTCATC-3’) and reverse (5’-AAACTGATTGCGAGAGAAG-3’)

primers. The amplification protocol had the following steps: 95°C for 30 s; 45 cycles of 95°C for 10 s, 60°C for 10 s and 72°C for 15 s; and cooling to 40°C for 40 s. Two housekeeping genes were used for data normalization: α-tubulin and elongation factor 1α (Protein IDs 24108 and 439881; http://genome.jgi-psf.org/Hebcy2/Hebcy2.home.html), using the primers described by Guerrero- Galán et al. (2018). Data analyses for gene expression levels were performed as described by Cuéllar et al. (2010). For comparisons among all means (n=4), a two-way ANOVA was performed followed by the Sidak's multiple comparison test. Differences at $P<0.05$ were considered significant.

1.4 *HcZnT2* cloning, plasmids and yeast strains

A cDNA library of *H. cylindrosporum* mycelia (collected from the symbiotic-interface mimicking assay) was used to amplify the *HcZnT2* full-length cDNA sequence. The amplification was carried out by PCR using the iProof High Fidelity DNA-polymerase (BioRad) and specific forward (5´-ATGAAGAGCACAACAAAGC-3´) and reverse (5´- TCAAGCAGCATCAACATC-3´) primers. For the expression of *HcZnT2* in yeast, the In-Fusion Cloning kit (Takara Bio) was used to clone the corresponding full-length cDNA into the pYES2 vector previously linearized with the *Hind*III and *Xba*I restriction enzymes, and the pYES2::*HcZnT2* construct was obtained. The resulting ligation reaction was transformed into *E. coli* TOP10, three independent clones were sequenced. For yeast expression of HcZnT2 fused to EGFP (Enhanced GFP), the stop codon was removed from the *HcZnT2* cDNA fragment and the yeast consensus sequence TACACA<u>ATG</u>TCT was included at the translation initiation site (underlined), with the TCT codon as a second triplet, in order to increase the efficiency of translation initiation, following manufacturer recommendations (Hamilton et al., 1987). The resulting fragment, the EGFP sequence and the *Hind*III-*Xba*I linearized pYES2 vector were fused with the help of the In-Fusion cloning Kit (Takara Bio) and the pYES::*HcZnT2-EGFP* construct was obtained.

For heterologous functional expression of HcZnT2 in yeast, the *S. cerevisiae* WT strain BY4741 (*MATa; his3Δ1; leu2Δ0; met15Δ0; ura3Δ0*), and three derived mutants were used: the Zn-hypersensitive double mutant strain *Δzrc1 Δcot1* (*MATa; his3Δ1; leu2Δ0; met15Δ0; ura3Δ0; zrc1::natMX3; cot1::kanMX4*), the Mn-hypersensitive mutant *Δpmr1* (*MATa his3Δ1 leu2Δ0 met15Δ0 ura3Δ0 PMR1::kanMX4*), and the Cd-hypersensitive mutant *Δycf1* (*MATa his3Δ1 leu2Δ0 met15Δ0 ura3Δ0 YCF1::kanMX4*) (EUROSCARF, Frankfurt, Germany). These three mutants and their parental WT strain BY4741 were co-transformed with either the pYES2 empty vector or the pYES2::*HcZnT2* (both of them ura+), together with the pYX222 empty vector (his+) using the LiAc/SS Carrier DNA/PEG method (Gietz and Woods, 2006). Systematic double transformation with the pYX222 (his+) empty vector was done to avoid addition of histidine in the culture medium, as histidine has been described as a Zn chelator (Krämer et al., 1996; Murphy et al., 2011). Transformed yeast were selected in synthetic defined medium lacking uracil and histidine [SD-His-Ura: 0,7% w/v yeast nitrogen base (Sigma), 2% D-glucose, and 0,075% w/v CSM-ura-his dropout amino acid supplement mixture (MP Biomedicals)]. For yeast subcellular localization, the HcZnT2-EGFP fusion was heterologously expressed in the *S. cerevisiae* WT strain BY4741 following the same procedure as described above.

1.5 Microscopy for subcellular localization in yeast

A Zeiss observer 7 fluorescence microscope equipped with an X-Cite 120 LED light source was used for yeast visualization. Three different filter sets allowed cell characterization of the green EGFP-labelled HcZnT2 protein (excitation BP 470/40, beam splitter FT 495, emission BP 525/50), the red FM4-64-labelled vacuolar membranes (excitation BP 545/25, beam splitter FT 570, emission BP 605/70) and the blue Hoescht 33342-labelled nuclei (excitation BP 325/390; beam splitter FT 395; emission BP 445/50). Images were taken with a Zeiss 63X Plan-Apochromat DIC 1.4 NA oil immersion objective and a monochrome Hammamatsu Orca Fllash 4 camera. Post-acquisition image processing was performed using ZEN lite 2012 software (Zeiss).

2 Supplementary Figures and Tables

2.1 Supplementary Figures

Supplementary Figure S1. Structural protein model of HcZnT2 using Alphafold.

Supplementary Figure S2. Growth of *Hebeloma cylindrosporum* in solid N6 media under different Zn conditions.

Supplementary Figure S3. Functional complementation of the cadmium-hypersensitivity of the *Δycf1* yeast mutant by HcZnT2.

2.2 Supplementary Tables

Supplementary Table S1. Functionally characterized CDF transporters of *Saccharomyces cerevisiae,* database used to retrieve the amino acid sequence, protein ID and transported substrate.

Supplementary Table S2. Selected fungal species included in this study, isolate/strain, database used to retrieve sequences, and consulted genome version.

Supplementary Figure S1. Structural protein model of HcZnT2 using Alphafold

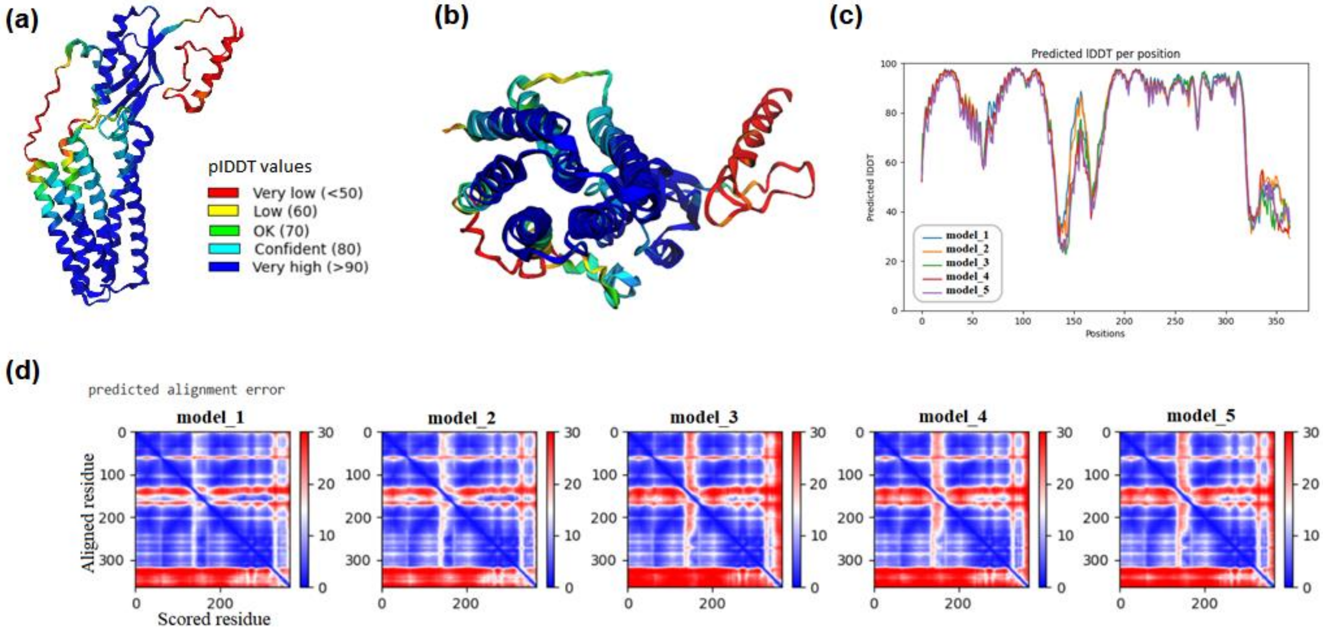

Supplementary Figure S1. Structural protein model of HcZnT2 using Alphafold. The side view (**a**) and top-down view (**b**) of the HcZnT2 consensus model computed with AlphaFold2 is shown, where residues are coloured depending on their predicted local distance difference test (plDDT) values. To evaluate the quality of the prediction, the plDDT values (**c**) and the Predicted Aligment Error (**d**) for the five generated HcZnT2 models are shown.

Supplementary Figure S2. Growth of *Hebeloma cylindrosporum* in solid N6 media under different Zn conditions

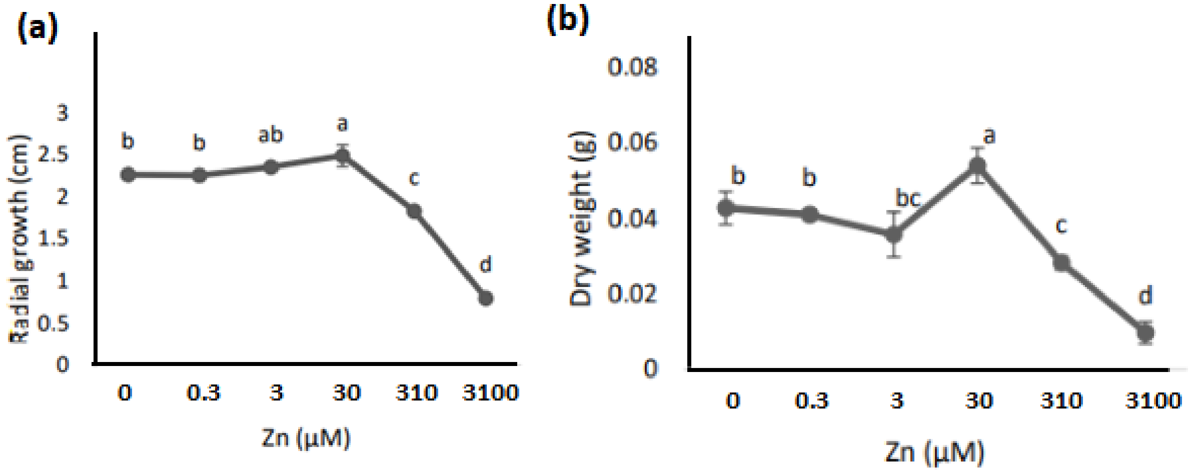

Supplementary Figure S2. Growth of *Hebeloma cylindrosporum* in solid N6 media under different Zn conditions. (**a**) Radial growth of *H. cylindrosporum* was measured by image J software, and (**b**) dry weight of the harvested fungus was analysed (n=4).

Supplementary Figure S3. Functional complementation of the cadmium-hypersensitivity of the *Δycf1* yeast mutant by HcZnT2

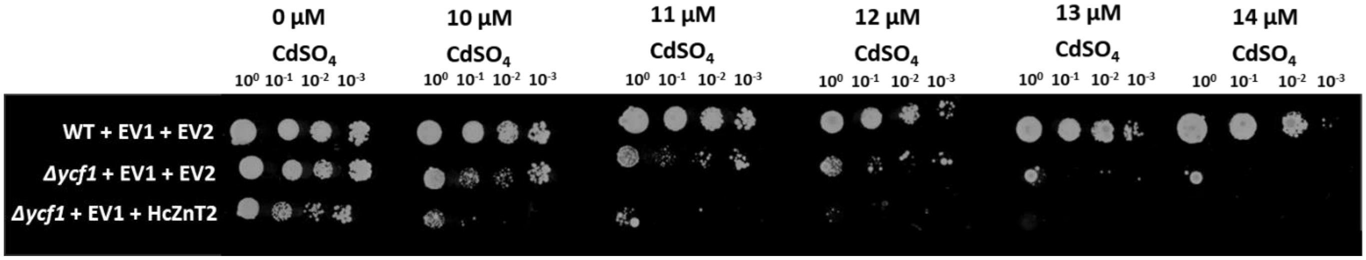

Supplementary Figure S3. Functional complementation of the cadmium-hypersensitivity of the *Δycf1* yeast mutant by HcZnT2. Wild-type BY4741 (WT) and mutant Δycf1 yeast strains harbouring the empty vector pYX222 (EV1) that brought histidine autotrophy and either pYES2 empty vector (EV2) or pYES2::HcZnT2 were used. Cultures with an OD_{600}=1 were 10-fold serial diluted (10^0, 10^{-1}, 10^{-2}, and 10^{-3}), as indicated above the panels. 10-µl-drops of these serial dilutions were spotted on SD-His-Ura induction control medium (0 µM $CdSO_4$) or the same medium supplemented with different concentrations of $CdSO_4$. Pictures were taken after 10 days of growth.

Supplementary Table S1

Supplementary Table S1. Functionally characterized CDF transporters of *Saccharomyces cerevisiae*, database used to retrieve the amino acid sequence, protein ID and transported substrate.

Name	Database	Protein ID	Substrate
**MSC2**	MycoCosm-JGI	1234	Zn
**ZRC1**	MycoCosm-JGI	4937	Zn
**COT1**	MycoCosm-JGI	5978	Zn
**ZRG17**	MycoCosm-JGI	5427	Zn
**MMT1**	MycoCosm-JGI	4865	Fe
**MMT2**	UniprotKB	Q08970	Fe

Supplementary Table S2. Selected fungal species included in this study, isolate/strain, database used to retrieve sequences, and consulted genome version.

Organism	Isolate/strain	Database	Version
Agaricus bisporus	H97	MycoCosm-JGI	V2.0
Amanita muscaria Koide	-	MycoCosm-JGI	V1.0
Auriscalpium vulgare	FP105234-Sp	MycoCosm-JGI	V1.0
Hebeloma cylindrosporum	h7	MycoCosm-JGI	V2.0
Heterobasidion annosum	-	MycoCosm-JGI	V2.0
Hydnomerulius pinastri	-	MycoCosm-JGI	V2.0
Laccaria bicolor	S238N	MycoCosm-JGI	V2.0
Lactarius quietus	S23C	MycoCosm-JGI	V1.0
Paxillus involutus	ATCC 200175	MycoCosm-JGI	V1.0
Peniophora sp.	-	MycoCosm-JGI	V1.0
Piloderma olivaceum	F1598	MycoCosm-JGI	V1.0
Pisolithus microcarpus	441	MycoCosm-JGI	V2.0
Rhizophagus irregularis	DAOM 197198	MycoCosm-JGI	V2.0

Russula atropurpurea	-	NCBI	-
Russula vinacea	BPL710	MycoCosm-JGI	V1.0
Saccharomyces cerevisiae	*ATCC 204508*	JGI & UniprotKB	-
Schizophyllum commune	H4-8	MycoCosm-JGI	V3.0
Schizosaccharomyces pombe	-	MycoCosm-JGI	-
Serpula lacrymans	S7.9	MycoCosm-JGI	V2.0
Stereum hirsutum	FP-91666 SS1	MycoCosm-JGI	V1.0
Suillus luteus	UH-Slu-Lm8-n1	MycoCosm-JGI	V3.0
